# Special Issue “Diabetes: Comorbidities, Therapeutics and Insights (2nd Edition)”

**DOI:** 10.3390/biomedicines13123037

**Published:** 2025-12-10

**Authors:** Tomislav Bulum

**Affiliations:** 1 Department of Diabetes and Endocrinology, Vuk Vrhovac University Clinic for Diabetes, Endocrinology and Metabolic Diseases, Merkur University Hospital, Dugi dol 4a, 10000 Zagreb, Croatia; tomobulum@gmail.com; Tel.: +38-512353887; Fax: +38-512331515; 2 School of Medicine, University of Zagreb, Šalata 3, 10000 Zagreb, Croatia

Although awareness of diabetes and its severe complications continues to grow, the disease remains one of the leading global causes of mortality and disability, regardless of age, gender, or geographic location [1]. In 2021, approximately 537 million people worldwide were affected by diabetes, and projections suggest that this number will rise to 783 million by 2045 [2]. Furthermore, the economic burden of diabetes—largely driven by the cost of managing its chronic complications—is expected to exceed USD 1054 billion by 2045 [3].

The most serious consequences of diabetes stem from its chronic complications, which are generally categorized into microvascular (diabetic kidney disease, retinopathy, and neuropathy) and macrovascular complications (primarily cardiovascular diseases) [4]. Diabetes remains the leading cause of blindness among the adult working-age population [5]. It is also the primary cause of chronic kidney disease, accounting for the majority of patients requiring dialysis and kidney transplantation [6]. Moreover, diabetes is the leading cause of non-traumatic lower limb amputations, with over 60% of such amputations occurring in individuals with diabetes [7]. Diabetic foot ulcers, often associated with both microvascular and macrovascular dysfunction, substantially increase the risk of limb loss [8].

While microvascular complications of diabetes are key contributors to long-term disability, macrovascular complications—including myocardial infarction, stroke, and peripheral arterial disease—are the predominant causes of premature morbidity and mortality in individuals with diabetes [9]. People with diabetes face a two- to four-fold higher risk of developing cardiovascular diseases, such as heart attacks and strokes, compared to those without diabetes, and they are significantly more susceptible to premature death [10]. This elevated cardiovascular risk is largely attributed to the clustering of metabolic risk factors commonly associated with obesity, such as hypertension, dyslipidemia, and chronic hyperglycemia. These risk factors are particularly prevalent in individuals with type 2 diabetes and are strongly linked to increased cardiovascular morbidity and mortality [10]. To underscore the interconnected nature of these conditions, the American Heart Association (AHA) recently introduced the concept of Cardiovascular-Kidney-Metabolic (CKM) syndrome, which highlights the integrated pathophysiology of cardiovascular, renal, and metabolic diseases [11]. Importantly, comprehensive risk factor management—beyond optimal glycemic control—remains essential. This includes strict regulation of blood pressure, lipid levels, body weight, and lifestyle factors such as smoking cessation, all of which are critical to reducing the burden of cardiovascular disease and lowering the risk of premature death in this high-risk population [12].

Sodium-glucose cotransporter 2 (SGLT2) inhibitors and glucagon-like-peptide-1 receptor (GLP-1) agonists represent new antidiabetic drugs that are now considered fundamental in the treatment of type 2 diabetes because of their favorable cardioprotective effects, including a decreased risk of diabetic nephropathy progression [13]. SGLT-2 inhibitors are particularly effective at reducing hospitalizations for heart failure and slowing the progression of kidney disease, while GLP-1 receptor agonists are well-known for reducing major adverse cardiovascular events. In addition, those new antidiabetic drugs have favorable effects on blood pressure, weight, and serum lipids that represent important metabolic risk factors implicated in the development and progression of micro and macrovascular complications in diabetes [13].

GLP-1 receptor agonists and partly SGLT-2 inhibitors can even prevent diabetes by helping prediabetic individuals revert to normal blood sugar levels, a process supported by weight loss and improved glycemic control. They increase insulin secretion, decrease glucagon, and reduce appetite, which aids in weight management and slows the progression to type 2 diabetes, especially in those who cannot tolerate metformin [14]. GLP-1 receptor agonists improve the quality of life for patients with type 2 diabetes and obesity through multiple mechanisms, including better glycemic control, significant weight loss, and reduced cardiovascular risk. They have also been associated with improved mental well-being, as demonstrated by reduced emotional eating and lower anxiety and depression scores in some studies, though side effects like gastrointestinal discomfort are common and can impact adherence [15]. For those on insulin therapy, digital health innovations for diabetes treatment include continuous glucose monitoring, connected insulin pens, automated insulin delivery systems, and telehealth. These technologies provide real-time data to patients and clinicians, improve self-management capabilities, and support more proactive and personalized care. Nowadays, one-third of individuals use mobile health applications for diabetic self-management, and more than half of individuals would like to manage their diabetes in the future by using mobile health applications [16].

Although recent trends indicate a decline in certain diabetes-related complications, such as all-cause and cardiovascular mortality, individuals with diabetes continue to face a significantly elevated risk of these outcomes compared to those without the disease [4]. Given this persistent disparity, further efforts are essential to reduce the burden of comorbidities and lower the risk of premature mortality in the diabetic population. In this context, this Special Issue brings together the latest research and advancements in the understanding and management of diabetes-related comorbidities, novel therapeutic approaches, and emerging clinical insights. It includes twelve original research articles, one review article, and one systematic review, offering a comprehensive perspective on current developments in the field.

The first research article in this Special Issue explored the increased risk of atrial fibrillation among individuals with diabetes. This study applied a clinical risk score derived from data obtained through the National Health and Nutrition Examination Survey (NHANES) to evaluate its predictive value for long-term outcomes and atrial fibrillation recurrence following catheter ablation. It outperformed the similarly structured C2HEST score by an average of five points. In addition, a plasma metabolomics analysis revealed three major metabolic features associated with an elevated risk of atrial fibrillation in patients with diabetes: disruption of energy metabolism, heightened inflammatory activity, and increased stress response [17].

The second research article examined how Croatian general practitioners (GPs) prescribe antidiabetic medications and apply treatment guidelines for type 2 diabetes. The survey results showed that around 59% of patients with type 2 diabetes are managed solely by GPs, with only 47% achieving target glycated hemoglobin levels. While nearly all GPs review therapy annually, only 47.6% fully understood the EASD/ADA guidelines; however, 58.3% consider both glycemic control and cardiovascular risk in their treatment decisions. The findings suggest that while outdated treatment approaches persist, Croatian GPs are increasingly adopting newer, guideline-recommended therapies [18,19].

The third research article evaluated the real-world effectiveness of minimally invasive revascularization in patients with diabetic foot ischemia, particularly focusing on device selection and lesion-specific predictors. The incidence of diabetic foot ulcers is continuously increasing, and a significant percentage of diabetic foot ulcers are caused by mixed micro- and macro-vascular dysfunction, leading to impaired perfusion of foot tissue [20]. In this study, among 98 patients (101 limbs), primary patency rates were 75.6% at 1 year and 67.6% at 2 years, with lower outcomes observed in those with chronic limb-threatening ischemia (CLTI). This study supports individualized treatment strategies and highlights the clinical benefit of drug-coated devices [21].

The fourth research article explored immunoglobulin G N-glycosylation in children with type 1 diabetes, focusing on differences between the Fc and Fab regions. Children at the onset of type 1 diabetes show altered N-glycosylation of plasma proteins and immunoglobulin G [22]. Using advanced ultra-performance liquid chromatography mass spectrometry (UPLC-MS) analysis, the researchers found that glycosylation changes in type 1 diabetes were significantly more pronounced in the Fab region than in the Fc. These findings suggest that early glycosylation changes in type 1 diabetes may originate primarily from the Fab region, potentially influencing immune modulation and autoimmunity [23].

The fifth research article investigated the relationship between genetic ancestry and pharmacogenetically relevant nucleotide allelic variants in 249 Mexican patients with type 2 diabetes. Using real-time PCR and ancestry-informative markers, researchers determined the proportions of Native American (65.5%), European (28.3%), and African (4.8%) ancestry in the cohort. Higher glycated hemoglobin levels were observed in patients with greater Native American ancestry, and uncontrolled diabetes was more common in individuals with higher Native American and lower European ancestry. These findings highlight that ethnicity is relevant for personalized medicine across different populations [24].

The sixth research article examined the prevalence and degree of malnutrition in patients with type 2 diabetes mellitus using the Controlling Nutritional Status (CONUT) and the Prognostic Nutritional Index (PNI) scores. A recently published meta-analysis reported that malnutrition affects approximately 33% of individuals with diabetes, while an additional 44% are considered at risk of developing malnutrition [25]. In this study, out of 266 patients, 64 met the inclusion criteria, with results showing that only 20.3% were well-nourished, while 67.2% had mild to moderate malnutrition. These findings highlight the significant burden of nutritional impairment in this population and underscore the need for routine nutritional assessment and intervention in diabetic care [26].

The seventh research article evaluated how changes in body weight influence clinical parameters in patients with obesity-related diabetes. A retrospective, longitudinal cohort study that included over 1 million people suggested that modest and sustained weight loss can lead to clinically meaningful improvements in glycemic and metabolic parameters among people with type 2 diabetes [27]. In this study, using medical records from Bucharest hospitals, the models showed strong predictive performance and demonstrated that weight loss significantly improved markers such as blood glucose, lipids, inflammation, liver enzymes, and blood pressure in patients with diabetes. These findings support weight management as a crucial strategy in diabetes care [28].

The eighth research article investigated the risk factors associated with early-onset diabetes, defined as diagnosis at ≤35 years, in Sindh, Pakistan. The prevalence of early-onset diabetes is rising globally and has more than doubled in men and tripled in women over recent decades [29]. This cross-sectional analysis of 754 patients identified several key predictors, including a strong family history of diabetes, sedentary lifestyle, frequent sugary beverage consumption, and abnormal sleep durations. These findings emphasize the importance of addressing modifiable lifestyle factors and conducting early screenings [30].

The ninth research article aimed to evaluate an artificial intelligence (AI)-based algorithm’s effectiveness in identifying patients with type 2 diabetes at risk for diabetic polyneuropathy, the most common diabetes-related complication. In recent years, AI has undergone remarkable advancements even in peripheral nervous system disorders [31]. In this study, in a sample of 201 patients, the AI algorithm categorized individuals into four risk groups, and its predictions were compared with biothesiometer measurements of vibratory perception threshold, a standard tool for diabetic polyneuropathy detection. About 31% of patients had abnormal vibratory perception threshold readings, indicating diabetic polyneuropathy. The findings suggest that AI tools may be valuable in triaging patients for further diagnostic evaluation, potentially improving early detection and management of diabetic polyneuropathy [32].

The tenth research article examined the relationship between GLP-1 and glucose-dependent insulinotropic polypeptide (GIP) receptor agonists, specifically semaglutide and tirzepatide, and the risk of vestibular disorders. These medications have revolutionized the treatment of type 2 diabetes and obesity, but their impact on vestibular function remains unclear [33]. This large retrospective cohort study analyzed data from over 496,000 patients using the TriNetX network to assess the risk of new-onset vestibular disorders following GLP-1 and GIP receptor agonist therapy. Both drugs were associated with a significantly increased risk of vestibular disorders compared to matched controls, with semaglutide users exhibiting a higher cumulative incidence than tirzepatide users [34].

The eleventh research article explored how alloxan-induced diabetes affects hormonal balance and oxidative stress in female Wistar rats. Diabetic rats showed a significant drop in estradiol and an increase in progesterone, indicating potential ovarian dysfunction. Elevated levels of transforming growth factor-beta 1 (TGF-β1) and glutathione peroxidase 3 (GPX3) pointed to heightened oxidative stress in diabetic animals. This study adds valuable insights into the metabolic and reproductive consequences of diabetes in female models [35].

The twelfth research article investigated the relationship between angiotensin-converting enzyme (ACE) and angiotensin II receptor type 1 (AGTR1) gene polymorphisms and hypertension, as well as the dipping pattern of blood pressure, in adolescents with type 1 diabetes. Several abnormal blood pressure patterns are frequently observed in individuals with type 1 diabetes and may signal the early development of diabetic complications [36,37]. In this study, which included 118 participants, those with hypertension were more often female and had a higher body mass index, higher triglyceride levels, greater insulin needs, and poorer glycemic control. The distribution of ACE rs1799752 and AGTR1 rs5186 genotypes did not significantly differ between hypertensive and normotensive groups, nor between dippers and non-dippers. Nearly half of the adolescents exhibited a non-dipping blood pressure pattern [38].

The first review deeply investigates pathogenesis and therapeutic perspectives of tubular injury in diabetic kidney disease. Diabetic kidney disease is a major microvascular complication of diabetes and the leading cause of end-stage kidney disease worldwide, affecting over one-third of type 1 and nearly half of type 2 diabetes patients [39]. Key mechanisms of tubular injury include metabolic disturbances, inflammation, cellular stress, epithelial–mesenchymal transition, impaired autophagy, and epigenetic changes leading to cellular senescence. In addition to traditional treatments like hypoglycemic and antihypertensive drugs, novel approaches such as stem cell therapy and gene editing are emerging. These advances offer promising new directions for diabetic kidney disease management beyond conventional therapies [40].

Finally, a systematic review evaluates the causal association between type 2 diabetes and Alzheimer’s disease. Type 2 diabetes mellitus has been linked to an increased risk of Alzheimer’s disease, but the causal nature of this relationship remains unclear [41]. This study combined a systematic review and meta-analysis of Mendelian randomization (MR) studies with a new two-sample MR analysis to clarify the association. Eight MR studies involving European populations were analyzed, and the pooled results showed no significant causal effect of type 2 diabetes on Alzheimer’s disease risk. A separate MR analysis using 512 genetic variants also found no significant association across multiple large Alzheimer’s disease datasets suggesting that genetic predisposition to type 2 diabetes does not causally increase Alzheimer’s disease risk [42].

In conclusion, this Special Issue offers a detailed overview of recent advances in the understanding of diabetes, its associated comorbidities, and current therapeutic strategies. This new knowledge will facilitate the diagnosis of these conditions and may be the basis for future therapeutic approaches. Overall, this Special Issue highlights the importance of continued research to improve outcomes for individuals living with diabetes.
